# A novel vaccine for mantle cell lymphoma based on targeting cyclin D1 to dendritic cells via CD40

**DOI:** 10.1186/s13045-015-0131-7

**Published:** 2015-04-14

**Authors:** Jingtao Chen, Gerard Zurawski, Sandy Zurawski, Zhiqing Wang, Keiko Akagawa, Sangkon Oh, Ueno Hideki, Joseph Fay, Jacques Banchereau, Wenru Song, A Karolina Palucka

**Affiliations:** Institute of Translational Medicine, the First Hospital, Jilin University, Changchun, 130031 China; Baylor Institute for Immunology Research and Sammons Cancer Center, Dallas, TX 75204 USA; The Present address: The Jackson Laboratory for Genomics Medicine, Farmington, CT USA; The Present address: AstraZeneca Pharmaceuticals LP, Gaithersburg, MD USA

**Keywords:** Mantle cell lymphoma, Cyclin D1, Vaccine, Dendritic cells, Tumor antigen

## Abstract

**Background:**

Mantle cell lymphoma (MCL) is a distinct clinical pathologic subtype of B cell non-Hodgkin’s lymphoma often associated with poor prognosis. New therapeutic approaches based on boosting anti-tumor immunity are needed. MCL is associated with overexpression of cyclin D1 thus rendering this molecule an interesting target for immunotherapy.

**Methods:**

We show here a novel strategy for the development of recombinant vaccines carrying cyclin D1 cancer antigens that can be targeted to dendritic cells (DCs) via CD40.

**Results:**

Healthy individuals and MCL patients have a broad repertoire of cyclin D1-specific CD4^+^ and CD8^+^ T cells. Cyclin D1-specific T cells secrete IFN-γ. DCs loaded with whole tumor cells or with selected peptides can elicit cyclin D1-specific CD8^+^ T cells that kill MCL tumor cells. We developed a recombinant vaccine based on targeting cyclin D1 antigen to human DCs via an anti-CD40 mAb. Targeting monocyte-derived human DCs *in vitro* with anti-CD40-cyclin D1 fusion protein expanded a broad repertoire of cyclin D1-specific CD4^+^ and CD8^+^ T cells.

**Conclusions:**

This study demonstrated that cyclin D1 represents a good target for immunotherapy and targeting cyclin D1 to DCs provides a new strategy for mantle cell lymphoma vaccine.

**Electronic supplementary material:**

The online version of this article (doi:10.1186/s13045-015-0131-7) contains supplementary material, which is available to authorized users.

## Introduction

Mantle cell lymphoma (MCL) is a distinct clinical subtype of B cell non-Hodgkin’s lymphoma (NHL) and accounts for approximately 5%–10% of all lymphoma cases. Current treatment is based on standard chemotherapy often combined with monoclonal antibody rituximab, followed by hematopoietic stem cell transplantation [1-3]. Although these treatment regimens can induce a high rate of remission, most patients ultimately relapse and cannot be cured [4,5]. Therefore, new therapeutic strategies are needed to improve the overall survival of patients and decrease treatment-associated morbidity.

A key common transforming event in the pathogenesis of MCL is chromosomal translocation t (11; 14) (q13; q32) leading to overexpression of cyclin D1. Cyclin D1 is a cell cycle regulator that is crucial for the G1-S transition. Its overexpression may facilitate the malignant transformation of the lymphoid cell and tumor progression, resulting in the deregulation of cell cycle control by inhibiting the suppressor effect of retinoblastoma 1 (RB1) and the cell cycle inhibitor p27 [6-8]. Although cyclin D1 negative cases have been reported [9-11], cyclin D1 overexpression still is considered a hallmark for MCL [12]. In addition to MCL, cyclin D1 has been detected in a wide variety of lymphoid and myeloid malignancies, including multiple myeloma, acute lymphoblastic leukemia, and hairy cell leukemia [13-15]. Also, it has been detected in other major malignancies, including colorectal, gastric, esophageal, lung, kidney, and breast cancer while little expression is found in normal tissues [16-21].

Several studies have investigated T cell responses to cyclin D1 and their potential use for immunotherapy [22-24]. Cyclin D1-specific cytotoxic T lymphocytes (CTLs) have been demonstrated in cancer patients with MCL and colorectal cancer [23,25,26]. CTLs specific for cyclin D1 were successfully generated from HLA-A2 positive healthy donors and MCL patients. These CTLs efficiently recognized target cells pulsed with their cognate peptide and cyclin D1 expressing tumor cell lines in an HLA-A*0201-restricted manner. More importantly, HLA-A*0201 matched, primary cyclin D1 positive tumor cells were efficiently recognized by cyclin D1-specific CTLs [26]. This suggests that cyclin D1 could be considered as a candidate antigen for immunotherapy despite our limited knowledge on the frequency and profile of cyclin D1-specific T cells in MCL patients.

Numerous approaches for the therapeutic vaccination of humans with cancer have been developed including autologous and allogeneic tumor cells (which are often modified to express various cytokines), peptides, proteins, and DNA vaccines (reviewed in [27]). *Ex vivo*-generated dendritic cells (DCs) have been used as therapeutic vaccines in patients with metastatic cancer for over a decade [28]. Importantly, a number of clinical studies have shown that DCs can expand T cells specific for non-mutated self-proteins that are overexpressed in cancer. The experimental success of using DC-specific antibodies to target antigens to individual DC subsets in conjunction with appropriately chosen adjuvant has appealing potential for the design of anti-cancer vaccines. Combined with a powerful adjuvant, vaccinating with one or multiple tumor-derived antigens coupled to DC-specific antibodies may amplify existing responses or break tolerance enabling the generation of protective responses. Studies to date demonstrate the targeting delivery of tumor antigens to DCs and Langerhan’s cells (LCs) [29] and the generation of therapeutic anti-tumor immunity [30] in animal models. More importantly, targeting both tumor and control antigens to human DCs *ex vivo* can lead to efficient antigen presentation and the subsequent generation of CD4^+^ T cell [31] and CD8^+^ T cell [32,33] responses. Furthermore, certain lectin receptors, including Dectin-1, LOX-1, and DC-SIGN, as well as other DC surface molecules (e.g., CD40), can provide additional activation signals to DCs [34-37].

Here, we have investigated specific T cell responses to the whole cyclin D1 protein, focusing on identifying potential dominant T cell epitopes. We found that both healthy individuals and MCL patients have a broad repertoire of cyclin D1-specific T cells thus supporting the utility of cyclin D1 as a tumor antigen for immunotherapy. Subsequently, we have developed a novel vaccine based on targeting cyclin D1 to DCs via the human DC surface receptor CD40 and explore the immune responses generated by this novel vaccine.

## Results

### Cyclin D1-specific IFN-γ secreting T cells in PBMCs from MCL patients

**Figure 1:**
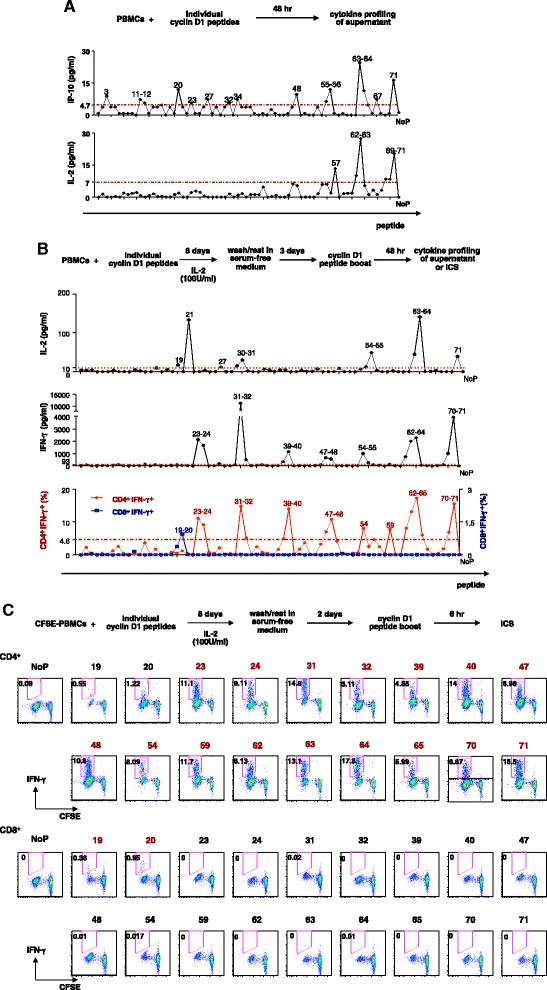
Mantle cell lymphoma patients display a broad repertoire of specific T cells to cyclin D1. PBMCs were isolated from a MCL patient (ACC-2000, HLAA* 02010101*3201, B*1501*3503, C*0303*1203, DRB1*0401*1401, DQB1*0503*0302), then 1 × 10^6^ cells per sample were stimulated with 71 individual 15-mer cyclin D1 peptides from Table 2. Median plus 5 multiplied median absolutedeviation (MAD) is considered a positive cutting line (shown as a red dash line). **(A)** Supernatants were harvested to test IP-10 and IL-2 secretion after 48-h co-culture. NoP is a no peptide negative control. **(B)** Supernatants of PBMCsafter 48-­h boosting were harvested to test cytokine IL-­2 and IFN-­γ secretion by Luminex®. The cells without peptide were used as negative control. Percentage of CD4^+^IFN-γ^+^ and CD8^+^IFN-γ^+^ population from IFN-­γ intracellular staining (from **(C)** was shown in a two-­line graph. **(C)** CFSE-labeled PBMCs were stimulated with cyclin D1 for 8 days and rested in serum-free medium for 2 days before boosting by the same peptide. Intracellular staining of IFN-γ was performed 6 h later. The cells without peptide were used as a negative control.

To assess the repertoire of cyclin D1-specific T cells, we investigated peripheral blood mononuclear cells (PBMCs) from five MCL patients (Table 1). A 15-mer overlapping peptide library (71 peptides) covering the whole protein was generated based on the cyclin D1 protein sequence (Table 2). PBMCs from patient ACC-2000 were stimulated with individual cyclin D1 peptides. Supernatants were harvested at 48 h, and cultures were continued for 8 days with IL-2 supplement (Figure 1A, B shows the scheme of experiment). At 48 h, we measured IL-2 and IP-10 secretion. As shown in Figure 1A, cytokine responses at 48 h were low with IP-10, nevertheless, peptide-specific peaks could be detected. These included 15 peptides (marked in the figure) inducing IP-10 production and six peptides inducing IL-2 secretion (Figure 1A).

**Table 1:** **Characterization of MCL patients**

**Patient ID number**	**Age**	**Gender**	**Prior Treatment**	**Ongoing Treatment**	**Disease Stage**	**HLA type**
1. ACC-2000, ACC-2003^a^	69	M	T, Chemo	-	Remission	A*0201B*1501*3503C*0303*1203 DRB1*0401*1401 DQB1*0503*0302
2. ACC-2038	54	M	Chemo	-	Remission	A*0201*2601B*3801*5101C*0701*1203 DRB1*1103*1301 DQB1*0301*0603
3. ACC-2805	58	M	Chemo	-	Remission	A*01*02 B*08*44 C* 05*07 *03(17)*07 DQB1*02
4. ACC-2501 ACC-2065^a^	57	M	T, Chemo	Chemo	Persistent	A*0101*0301 B*4402 C* 0501 DRB1*0401*1501 DQB1*0301*0602
5. ACC-2781	66	M	No treatment	-	Just diagnosis	A*01*31 B*08*40 (60) C*03*07 DRB1*03*04 DQB1*02*0302

*T* transplant, *Chemo* chemotherapy.

All the MCL patients are Caucasian.

^a^Patients 1 and 4 had two blood draws indicated with different patient ID.

**Table 2:** **15-mer cyclin D1 overlapping library**

**Peptide**	**Position**	**Sequence**	**Peptide**	**Position**	**Sequence**	**Peptide**	**Position**	**Sequence**
1	1–15	MEHQLLCCEVETIRR	26	101–115	LLGATCMFVASKMKE	51	201–215	SMVAAGSVVAAVQGL
2	5–19	LLCCEVETIRRAYPD	27	105–119	TCMFVASKMKETIPL	52	205–219	AGSVVAAVQGLNLRS
3	9–23	EVETIRRAYPDANLL	28	109–123	VASKMKETIPLTAEK	53	209–223	VAAVQGLNLRSPNNF
4	13–27	IRRAYPDANLLNDRV	29	113–127	MKETIPLTAEKLCIY	54	213–227	QGLNLRSPNNFLSYY
5	17–31	YPDANLLNDRVLRAM	30	117–131	IPLTAEKLCIYTDNS	55	217–231	LRSPNNFLSYYRLTR
6	21–35	NLLNDRVLRAMLKAE	31	121–135	AEKLCIYTDNSIRPE	56	221–235	NNFLSYYRLTRFLSR
7	25–39	DRVLRAMLKAEETCA	32	125–139	CIYTDNSIRPEELLQ	57	225–239	SYYRLTRFLSRVIKC
8	29–43	RAMLKAEETCAPSVS	33	129–143	DNSIRPEELLQMELL	58	229–243	LTRFLSRVIKCDPDC
9	33–47	KAEETCAPSVSYFKC	34	133–147	RPEELLQMELLLVNK	59	233–247	LSRVIKCDPDCLRAC
10	37–51	TCAPSVSYFKCVQKE	35	137–151	LLQMELLLVNKLKWN	60	237–251	IKCDPDCLRACQEQI
11	41–55	SVSYFKCVQKEVLPS	36	141–155	ELLLVNKLKWNLAAM	61	241–255	PDCLRACQEQIEALL
12	45–59	FKCVQKEVLPSMRKI	37	145–159	VNKLKWNLAAMTPHD	62	245–259	RACQEQIEALLESSL
13	49–63	QKEVLPSMRKIVATW	38	149–163	KWNLAAMTPHDFIEH	63	249–263	EQIEALLESSLRQAQ
14	53–67	LPSMRKIVATWMLEV	39	153–167	AAMTPHDFIEHFLSK	64	253–267	ALLESSLRQAQQNMD
15	57–71	RKIVATWMLEVCEEQ	40	157–171	PHDFIEHFLSKMPEA	65	257–271	SSLRQAQQNMDPKAA
16	61–75	ATWMLEVCEEQKCEE	41	161–175	IEHFLSKMPEAEENK	66	261–275	QAQQNMDPKAAEEEE
17	65–79	LEVCEEQKCEEEVFP	42	165–179	LSKMPEAEENKQIIR	67	265–279	NMDPKAAEEEEEEEE
18	69–83	EEQKCEEEVFPLAMN	43	169–183	PEAEENKQIIRKHAQ	68	269–283	KAAEEEEEEEEEVDL
19	73–87	CEEEVFPLAMNYLDR	44	173–187	ENKQIIRKHAQTFVA	69	273–287	EEEEEEEEVDLACTP
20	77–91	VFPLAMNYLDRFLSL	45	177–191	IIRKHAQTFVALCAT	70	277–291	EEEEVDLACTPTDVR
21	84–95	AMNYLDRFLSLEPVK	46	181–195	HAQTFVALCATDVKF	71	281–295	VDLACTPTDVRDVDI
22	85–99	LDRFLSLEPVKKSRL	47	185–199	FVALCATDVKFISNP			
23	89–103	LSLEPVKKSRLQLLG	48	189–203	CATDVKFISNPPSMV			
24	93–107	PVKKSRLQLLGATCM	49	193–207	VKFISNPPSMVAAGS			
25	97–111	SRLQLLGATCMFVAS	50	197–211	SNPPSMVAAGSVVAA

At day 8 of culture, the cells were rested for 2 days and restimulated for 48 h to analyze peptide-specific cytokine responses. As shown in Figure 1B, 14/71 peptides elicited strong IFN-γ response with up to 1 ng/ml IFN-γ secreted in response to peptide 31. IL-2 was produced in response to ten peptides (Figure 1B).

**Figure 2:**
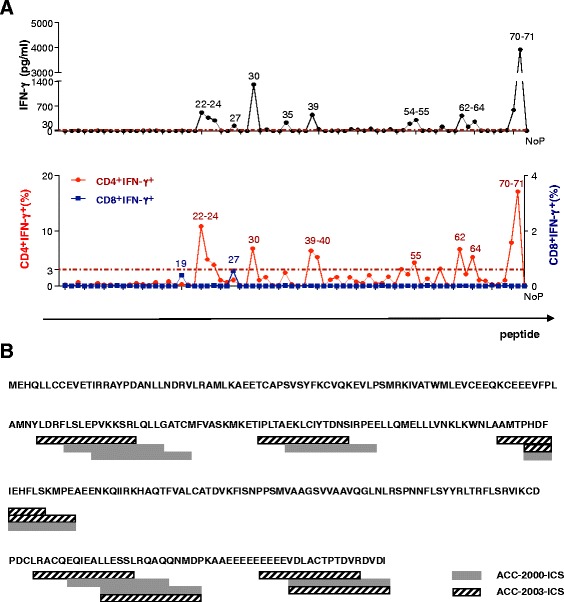
Long live of specific T cells to cyclin D1. **(A)** Another blood draw 3 months later from the MCL patient ACC-­2000, indicated as ACC-­2003. IFN-­γ cytokine secretion and percentage of CD4^+^IFN-γ^+^ and CD8^+^IFN-γ^+^ population from IFN-γ intracellular staining were shown. MAD is considered a positive cutting line (shown as a red dash line). **(B)** The potential cyclin D1 epitopes that stimulate CD4^+^T cells to produce IFN-­γ based on intracellular staining data are indicated on the protein sequence of cyclin D1. Data shown are two independent experiments by using PBMCs from two blood draws of the same MCL patient.

Next, we wanted to analyze the frequency and type of T cells specific to cyclin D1. CFSE-labeled PBMCs from patient ACC-2000 were cultured with cyclin D1 peptides, restimulated at day 11 with respective peptides, and cytokine profiles were measured using multicolor intracellular cytokine assay (ICS) (Figure 1C). Remarkably, 16/71 of the cyclin D1 peptides induced intracellular IFN-γ expression by CD4^+^ T cells (Figure 1C). This suggests the presence of cyclin D1-specific Th1 cells in MCL patients. Two out of 71 cyclin D1 peptides also induced intracellular IFN-γ expression by CD8^+^ T cells (Figure 1C). The peptides that could stimulate CD4^+^ and CD8^+^ T cells were different (Figure 1C). ICS data were further confirmed by the analysis of peptide-specific cytokine responses assessed in the supernatants of cultures restimulated for 48 h. There, a number of peptides were able to elicit IFN-γ secretion (Figure 1C). Next, Luminex® results reflecting the IFN-γ secretion into supernatants were overlaid with ICS results reflecting the phenotype of IFN-γ secreting T cells (Figure 1B). This analysis clearly indicated that CD4^+^ and CD8^+^ T cells recognize different cyclin D1 epitopes, and that CD4^+^ T cell repertoire is much broader than that of CD8^+^ T cells. The analysis of PBMCs from the same patient from a second blood draw ACC-2003 obtained 3 months later showed the same repertoire of IFN-γ secreting T cells (Figure 2A, B). Thus, cyclin D1-specific T cell immunity in MCL patients may be long lived.

### Cyclin D1-specific T cells in a cohort of MCL patients

**Figure 3:**
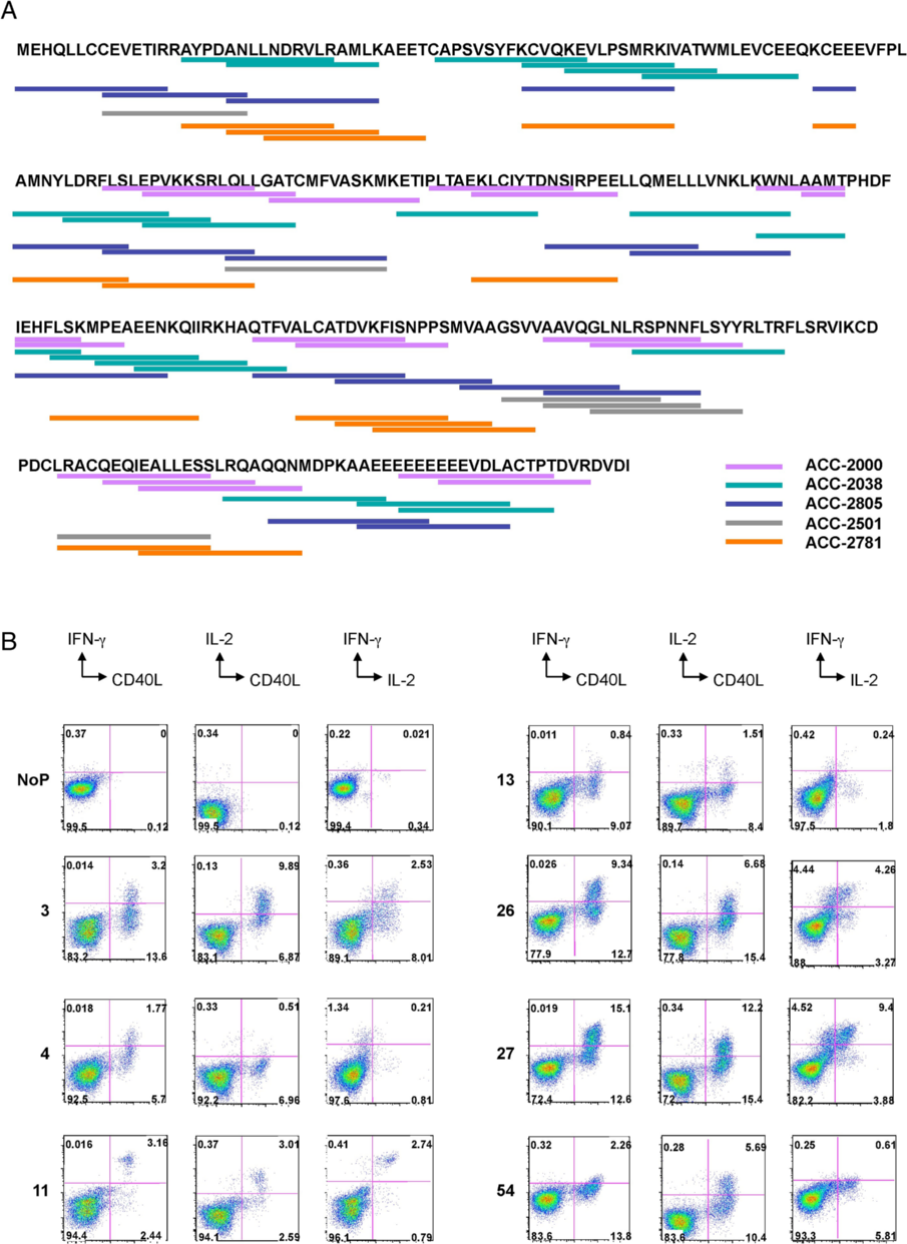
Cytokine profiles from different MCL patients**. (A)** The potential cyclin D1 epitopes that induced IFN-γ production in PBMCs obtained from five MCL patients on protein sequence of cyclin D1, based on intracellular staining and Luminex® data. **(B)** Intracellular staining of IFN-γ, IL-2, and CD40L were performed on a MCL patient ACC-2805 (HLA-A*01*02 B*08*44 C*05*07 DRB1*03*07 DQB1*02). The expressions from CD4^+^ gated were shown. Similar patterns were found in other two patients.

Having identified a broad repertoire of cyclin D1-specific IFN-γ T cells in the PBMCs of one patient, we next analyzed PBMCs from a cohort of four additional MCL patients (Table 1). We used Luminex®-based cytokine profiling upon PBMC restimulation as illustrated in Figure 1B. As shown in Figure 3A, PBMCs from all five analyzed patients displayed cyclin D1-specific secretion of IFN-γ. PBMCs from most of the patients showed responses to a larger number of peptides (>10) except PBMCs from the patient ACC-2501. While such differences might be related to the precursor frequency and/or might be driven by ongoing treatment; nevertheless, this analysis demonstrates that MCL patients have circulating memory T cells specific to cyclin D1. Thus, both intracellular staining and secreted cytokine productions established the level of cyclin D1-specific T cells induced by cyclin D1 peptides (Figure 3B). The secretion of cytokines by antigen-specific T cells was further confirmed by ICS in combination with CD154 staining. As illustrated in Figure 3B, antigen-specific CD154^+^ CD4^+^ T cells secreted IFN-γ and IL-2 in the PBMCs from patient ACC-2805. Similar patterns were found in other patients. In addition, the analysis of PBMCs from five healthy donors (Additional file 1: Table S1) revealed sporadic detection of cyclin D1-specific T cell responses (Additional file 2: Figure S1). Taken together, these results indicate the presence of a broader repertoire of cyclin D1-specific T cells in MCL patients and healthy donors.

### Selection of HLA-A*0201 binding cyclin D1 CD8^+^ T cell epitopes

Despite detecting cyclin D1-specific T cells, our analysis, thus far, showed a broad CD4^+^ T cell repertoire but a rather narrow CD8^+^ T cell repertoire in MCL patients. This could be due to the length of the peptides as class I-dependent CD8^+^ cells respond better to 8-12-mers. Because CTLs are a major effector arm of anti-tumor immunity, we analyzed in greater detail on the induction of cyclin D1-specific CD8^+^ T cells from PBMCs of healthy donors. To this end, we selected cyclin D1 peptides which could induce strong IFN-γ production as described above and then screened the cyclin D1 sequences for their binding affinity to HLA-A*0201 using peptide-binding databases (http://www.immuneepitope.org). Table 3 shows the sequence of human cyclin D1 peptides that could potentially bind to HLA-A*0201 as deduced by computational predictive binding scores.

**Table 3:** Potential cyclin D1 peptides for HLA-A*0201 molecules

**Name**	**Sequence**	**Length (mer)**	**Predictive binding score** ^**a**^
P5-13	LLCCEVETI	9	6.0
P63-71	WMLEVVCEEQ	9	947.8
P151-159	NLAAMTPHD	9	743.7
P202-210	MVAAGSVVA	9	854.6
P204-212	AAGSVVAAV	9	703.7
P4-13	QLLCCEVETI	10	133.6
P21-30	NLLNDRVLRA	10	324.3
P58-67	KIVATWMLEV	10	255.9
P100-109	QLLGATCMFV	10	8.3
P101-110	LLGATCMFVA	10	184.0
P123-132	KLCIYTDNSI	10	246.9
P154-163	AMTPHDFIEH	10	981.0
P164-173	FLSKMPEAEE	10	508.5
P203-212	VAAGAVVAAV	10	580.3
P253-262	ALLESSLRQA	10	474.4
P57-67	RKIVATWMLEV	11	354.3
P94-104	VKKSRLQLLGA	11	582.3
P99-109	LQLLGATCMFV	11	711.1
P106-116	CMFVASKMKET	11	339.2
P123-133	KLCIYTDNSIR	11	362.3
P154-164	AMTPHDFIEHF	11	904.8
P158-168	HAFIEHFLSKM	11	577.7
P202-212	MVAAGSVVAAV	11	746.2
P208-218	VVAAVQGLNLR	11	442.9

^a^Predictions (ann methods)—low IC50 values (nM) = good binder.

**Figure 4:**
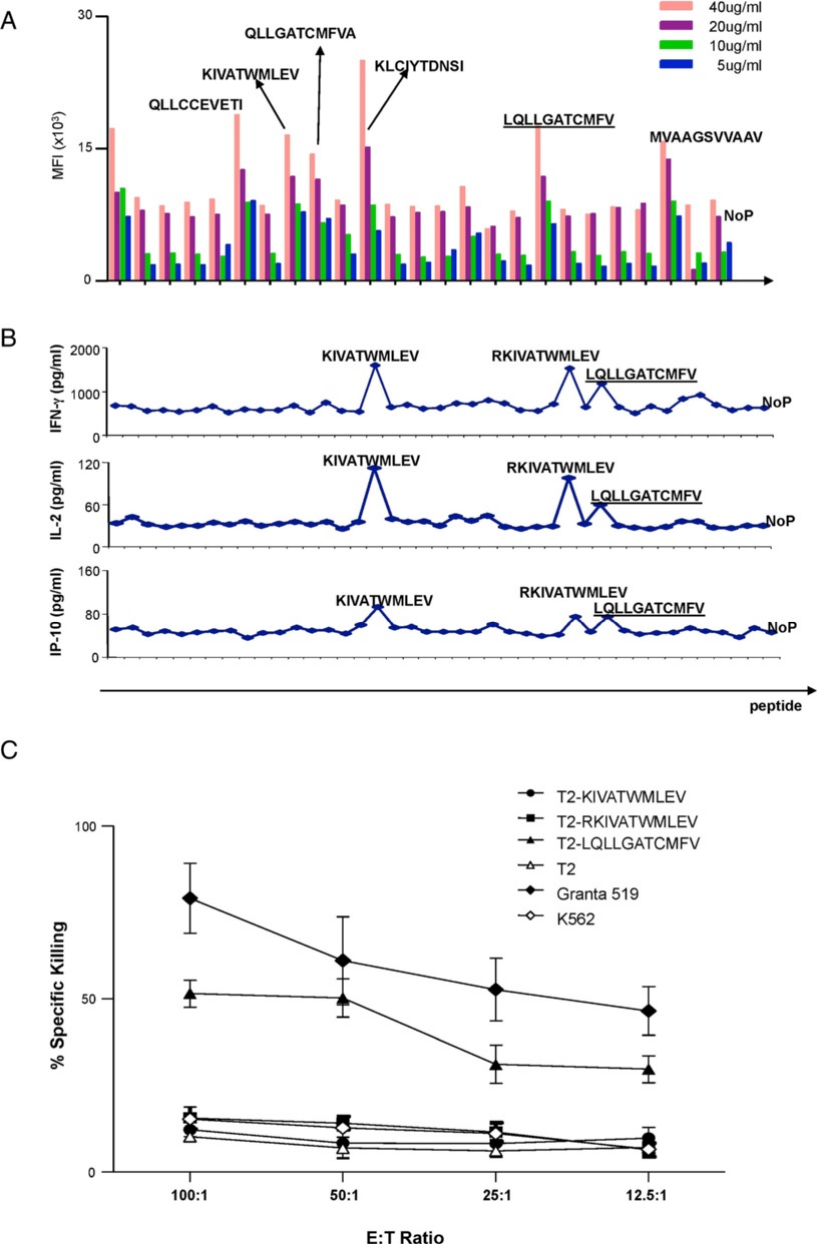
Identifying dominant HLA-A*0201-restricted cyclin D1 T cell epitopes. **(A)** Binding capacity for cyclin D1 peptides to HLA-A*0201 molecules on T2 cells. T2 cells were cultured with predicted cyclin D1 peptides which have high binding affinity to HLAAA* 0201 (list in Table 3), in RPMI1604 without FCS for 18 h, and subsequently stained for cell surface expression of MHC class I. Up-regulation of HLA-A2 expression after binding with specific peptides was represented as median fluorescence intensity (MFI). Sequences of positive peptides are shown. **(B)** IFN-DCs primed by the individual cyclin D1 peptide as (A) were co-cultured with enriched CD8^+^ T cells for 10 days. Then, cells were boosted with the same peptide pulsed T2 cells at 37°C for 36 h, thereafter, supernatant was harvested, and the cytokines production were tested by Luminex®. Sequences of positive peptides are shown. **(C)** Enriched CD8^+^ T cells from a healthy donor ND239 (HLA-A*0201) were stimulated with autologous IFΝ-DCs pulsed with MCL lymphoma cell dying bodies (Granta 519) and treated with LPS for 6 h. Ten days later, induced specific CTLs were tested in a standard 4-h ^51^Cr release assay. Target cells used were cyclin D1 peptide P_58–67_ KIVATWMLEV-pulsed T2 cells, P_57–67_ RKIVATWMLEV-pulsed T2 cells, P_99–109_ QLLGATCMFV-pulsed T2 cells, non-pulsed T2 cells, cyclin D1^+^HLA-A*0201^+^ MCL lymphoma cell line Granta 519, and K562 as natural killing activity controls.

The binding affinity of cyclin D1 peptides (Table 3) to HLA-A*0201 molecules was determined using the HLA-A2 TAP-deficient T2 lymphoma cell line, which enhances HLA-A2 expression when exposed to exogenous HLA-A2-binding peptides. This assay confirmed that P_4–13_, P_58–67_, P_100–109_, P_123–132_, P_99–109_, and P_202–212_ can bind HLA-A*0201 molecules (Figure 4A). To test CD8^+^ T cell responses, CD8^+^ T cells from a healthy donor (ND239 HLA-A*0201) were expanded by stimulating with DCs loaded with cyclin D1 peptides. Then, T cells were restimulated with peptide-pulsed T2 cells at 37°C for 36 h to test the cytokine production in the supernatant by Luminex®. As shown in Figure 3B, among the seven cyclin D1 peptides able to bind HLA-A*0201 on T2 cells, only P_58–67_ (KIVATWMLEV), P_57–67_ (RKIVATWMLEV), and P_99–109_ (LQLLGATCMEV) could induce cytokine production. Cells pulsed with no peptide were used as negative controls in binding affinity studies and the cytokine release assay (Figure 4A, B). Thus, three cyclin D1 peptides P_58–67_, P_57–67_, and P_99–109_ could induce potent CD8^+^ T cell responses.

Next, we assessed whether the identified CD8^+^ T cell epitopes can be cross-presented to elicit specific CTLs. There, enriched HLA-A*0201^+^ CD8^+^ T cells were expanded by GM-CFS/IFN-DCs pulsed with MCL cell line Granta 519 dying bodies. After a single round of stimulation and 10-day culture, the T cells were tested for their capacity to kill cyclin D1-expressing target cells using a standard ^51^Cr-release assay. Figure 4C shows that CD8^+^ T cells could kill Granta 519 MCL cells that were used as the antigenic cargo to load the DCs. Control K562 cells were not killed suggesting CTL lysis. This was further confirmed by the capacity of elicited CTLs to kill T2 cells pulsed with cyclin D1 peptide P_99–109_ (Figure 4C). Thus, this peptide can be cross-presented and recognized by CD8^+^ T cells. Though cyclin D1 peptides P_58–67_ and P_57–67_ were able to induce cytokine secretion (Figure 4A, B), no killing was observed for these two individual peptides loaded on T2 cells (Figure 4C).

### Recombinant fusion protein anti-CD40-cyclin D1 efficiently expands specific CD8^+^ T cells

**Figure 5:**
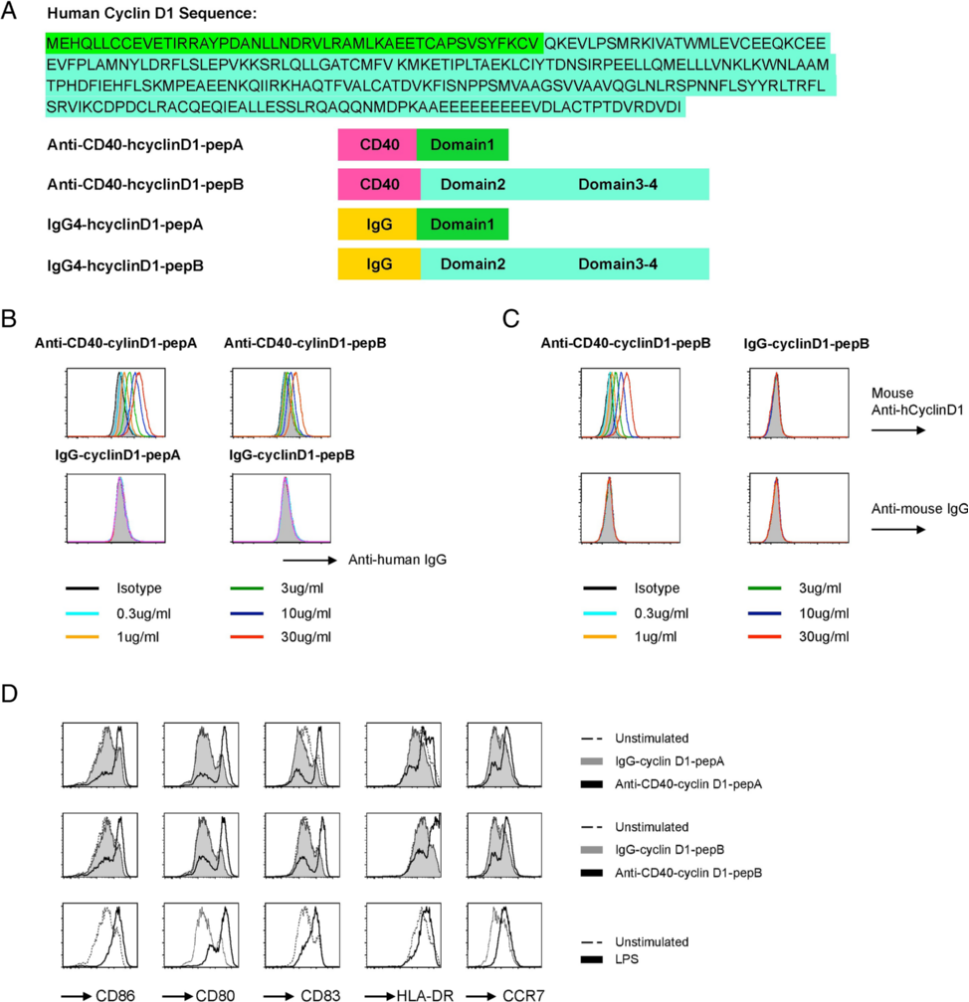
Characterization of recombinant cyclin D1 fusion proteins.** (A)** The construction of cyclin D1 fused to DC receptor CD40 recombinant IgG4 mAb or non-DC binding IgG4 as a control. The sequence of the different human cyclin D1 protein domains is shown in different colors. **(B, C)** Anti-CD40-cyclin D1 Abs detected on the surface of monocytederived IFN-DCs. Flow cytometry staining of IFN-DCs with anti-human IgG (B), antihuman cyclin D1 (C), or anti-mouse IgG isotype control mAbs **(C)**. **(D)** The expression of several molecules (CD86, CD80, CD83, HLA-DR, and CCR7) on the IFN-DCs was significantly increased after co-culture with anti-CD40-cyclin D1 fusion proteins for 48 h, compared with co-culture with IgG4-cyclin D1 control proteins. The data from a representative of three independent experiments are shown; different donors showed similar results.

CD40 is a co-stimulatory molecule belonging to the tumor necrosis factor receptor family and is expressed on many cells including DCs, monocytes, and B cells [38,39]. Anti-CD40 mAbs were able to facilitate the maturation of DCs, and DCs mediated T cell activation [40-42]. Delivery of antigen via mAb to CD40 has been shown to induce antigen-specific immune responses and provide protection against cancer [43], as well as control HIV infection *in vitro* [44]. Thus, to explore the potential of this novel vaccine, large cyclin D1 domains were fused to the heavy chain of anti-CD40 Abs (anti-CD40-cyclin D1 mAb) along with isotype control, IgG4 mAbs. Figure 5A shows the construction of these fusion proteins. Domain 1 was fused to DC receptor CD40 or isotype control IgG4, generating anti-CD40-cyclin D1-pepA and IgG4-cyclin D1-pepA protein. Domains 2, 3, and 4 were fused to DC receptor CD40 or isotype control IgG4, generating anti-CD40-cyclin D1-pepB and IgG4-cyclin D1-pepB protein. Together, these two anti-CD40 fusion proteins carried the entire cyclin D1 sequence.

We next tested whether cyclin D1 could be presented to the DC surface by the fusion proteins. GM-CSF/IFN alpha monocyte-derived DCs (IFN-DCs) were first incubated with fusion proteins for 30 min on ice to prevent internalization, cyclin D1 presented on the surface of DCs was detected by anti-human IgG Abs (Figure 5B), and confirmed by using anti-human cyclin D1 Ab (Figure 5C). Anti-human-cyclin D1 mAb (clone: G124-326) recognized anti-CD40-cyclin D1-pepB, but not anti-CD40-cyclin D1-pepA, IgG4-cyclin D1-pepA, and IgG4-cyclin D1-pepB (Figure 5C). Anti-human-cyclin D1 mAb is a monoclonal antibody, which recognized full length cyclin D1, so it may not identify the short part of cyclin D1 presented by anti-CD40-cyclin D1-pepA. Isotype control IgG4-cyclin D1-pepA and IgG4-cyclin D1-pepB could not present to the DC surface.

In addition, the expression of activation markers and co-stimulatory molecules (CD83, CD86, CD80, HLA-DR, and CCR7) on IFN-DCs was significantly increased by 48 h after co-culture with anti-CD40-cyclin D1 Abs (Figure 5D). This data demonstrated the activating properties of recombinant anti-CD40-cyclin D1 fusion proteins compared to the matching IgG4 control fusion proteins.

**Figure 6:**
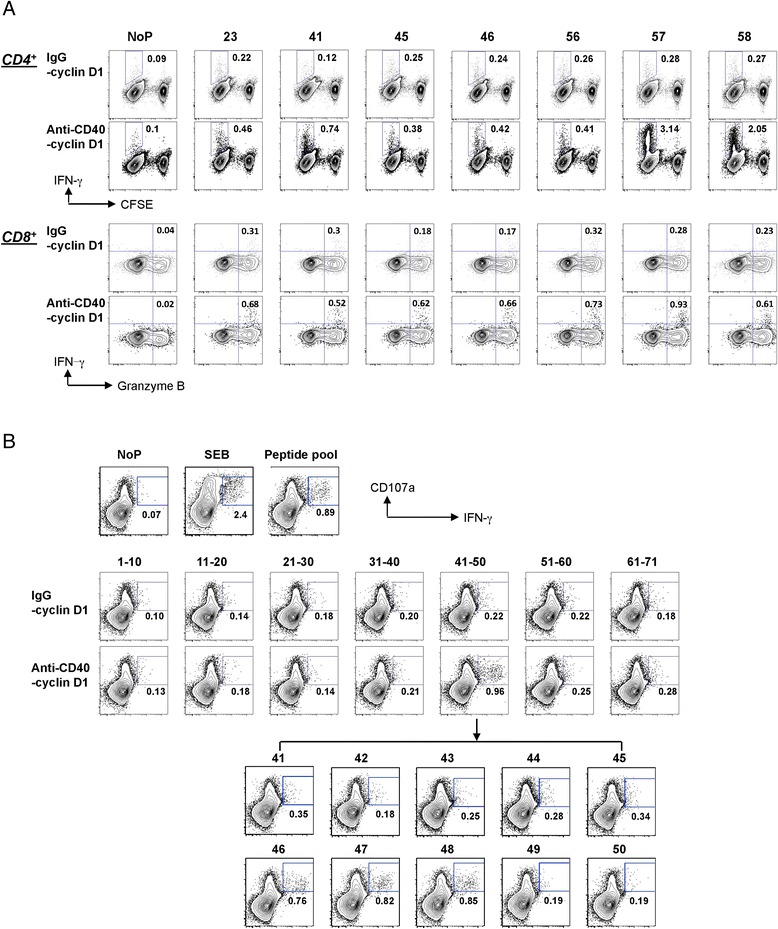
Targeting cyclin D1 to DCs via CD40 elicits cyclin D1-specific CD4^+^ and CD8^+^T cell responses. **(A)** 5 x10^3^ IFN-DCs were loaded with 3 μg/ml recombinant cyclin D1 fusion proteins or control IgG-cyclin D1. After 8 h, CFSE-labeled autologous enriched CD3^+^T cells were co-cultured for 7 days then added the same amount of cyclin D1 fusion protein or control IgG-cyclin D1-loaded IFN-DCs cultured for another 7 days. Cells were boosted by 71 of 15-mer cyclin D1 peptides following 2-day resting in serum-free medium. Intracellular staining of IFN-γ was performed 8 h later. The cells without peptide boosting were used as a negative control. The percentage of IFN-γ^+^CFSE^−^ cells in gated CD4^+^ T cells and IFN-γ^+^Granzyme B^+^ cells in gated CD8^+^ T cells were indicated. This data is a representative of three independent experiments of a healthy donor ND257 (HLA-A*0201). **(B)** Intracellular staining of IFN-γ/CD107a performed in another healthy donor ND239 (HLA-A*0201) was shown. Percentage of IFN-γ^+^CD107a^+^ cells from CD8^+^ gated is indicated.

Subsequently, the targeting ability of CD40-cyclin D1 recombinant fusion protein to DCs was investigated for the capacity to activate cyclin D1-specific T cells from healthy individuals. IFN-DCs targeted with anti-CD40-cyclin D1 mAb expanded a broad repertoire of cyclin D1-specific CD4^+^ and CD8^+^ T cells (Figure 6A, B).

To examine the cytolytic capability of cyclin D1-specific CD8^+^ T cells, we assessed the functional capacity of prototype vaccine-expanded CD8^+^ T cells to produce effector cytokines, cytolytic factors, and degranulation capacity as determined by externalization of CD107a. IFN-DC presented anti-CD40-Cyclin D1 to T cell cultures from a healthy donor; in response to peptide challenge, cyclin D1-specific CD8^+^ T cells positive for CD107a and granzyme B with IFN-γ were induced (Figure 6A, B).

Collectively, these data demonstrate the capacity of anti-CD40-cyclin D1 recombinant fusion proteins to expand cyclin D1-specific CD4^+^ and CD8^+^ T cells. Currently, the anti-CD40-cyclin D1 recombinant vaccine is being tested *in vivo* in non-human primates. This strategy will facilitate the development of a mantle cell lymphoma vaccine.

## Discussion

The better understanding of anti-tumor immune response and tumor immune escape mechanisms and the exploration of new ways for different effects and mechanisms of tumor immunotherapy and immunotherapy will facilitate new and innovative approaches to human tumor immunotherapy. Immunotherapy is moving to the vanguard of cancer therapy. Cancer immunotherapy is being increasingly used to drive the immune system to treat tumors [45], and tumor antigens are the most appropriate targets for cancer immunotherapy [46]. The antigen of interest can be used to vaccinate as a whole protein or with synthetic peptides derived from this protein. Presentation of T cell epitopes on MHC complexes can successfully induce T cell responses. T cells specific to subdominant epitopes have been shown to participate in anti-tumor immune responses [47].

The first clinical trial of a melanoma antigen gene-1 (MAGE-1)-derived peptide-based vaccine was reported in 1996 [48]. Afterward, numerous clinical trials of peptide vaccines have been carried out to assess the ability of these vaccines to induce clinical responses in different cancer patients, and some promising clinical responses have been observed. A number have already received FDA approval, including a personalized peptide vaccination protocol [49]. Peptides recognized by CTLs or helper T cells are generally derived from fragments of tumor antigen proteins, and an increasing variety of non-classical events were shown to contribute to the production of these peptides [50]. A database containing human antigenic peptides which aims to guide scientists and clinicians searching for appropriate cancer vaccine candidates is available and is constantly being updated [46].

Here, we expanded cyclin D1-specific IFN-γ secreting T cells in PBMCs from MCL patients, as well as from healthy donors. A number of cyclin D1 peptides were able to stimulate IFN-γ production and showed a broad CD4^+^ T cell repertoire but a narrow CD8^+^ T cell repertoire. To do more analysis, crucial for an effective vaccine therapy, we screened peptides based on MHC-binding algorithms and cytokine secretion. Three cyclin D1 peptides P_58–67_, P_57–67_, and P_99–109_ induced potent CD8^+^ T cell responses. One of these peptides, P_99–109_, could be cross-presented and recognized by CD8^+^ T cells. In accord with our results, HLA-A*0201-binding cyclin D1 epitopes were also previously reported [23,24,26]. The HLA-DR4-restricted T cell epitope P_198–212_: NPPSMVAAGSVVAAV derived from cyclin D1 epitope was identified by mass spectrometry [22]. Thus, our results highlight the importance of verifying the functional peptide sequences in vaccines. The finding that immune reactivity against cyclin D1 was also found in healthy donors could mean that cancer patients have a high frequency of cyclin D1-specific T cell precursors in the blood, potentially leading to a higher efficacy of cyclin D1-targeted anti-tumor vaccination.

Dendritic cells (DCs) are specialized in antigen processing and presentation. DC-based experimental cancer vaccines have shown some success in patients with lymphoma and other cancers. Numerous receptors are expressed on DCs, including three categories: receptor kinases, toll-like receptors (TLRs), and C-type lectin receptors. By targeting these DC receptors, a more competent approach of delivering antigens in DC-based anti-cancer immunotherapy is becoming a promising vaccination strategy. The specific targeting of antigens to DCs *in vivo* could enhance potent antigen-specific CD4^+^ and CD8^+^ T cell-mediated immunity [51-53]. DC targeting not only assists the delivery of an antigen but also potentially provides an activation signal by targeting activating DC receptor antibodies [45].

In this context, cyclin D1 is a promising tumor-associated antigen (TAA) for MCL. It is consistently overexpressed in virtually all MCL patients. Moreover, the presence of cyclin D1-specific CD8^+^ T cells in MCL patients is proven. Our previous study using anti-CD40.HIV5pep antibody, which has a physical linkage between the five long HIV peptides from Gag, Nef, and Pol with the CD40-targeting antibody, could also induce HIV-specific T cells *in vitro* [44]. In order to develop a specific immune response against MCL, recombinant cyclin D1 antigen carried by an anti-DC receptor vehicle CD40 was delivered to IFN-DCs for MHC class-I cross-presentation in T cell co-cultures. This resulted in the expansion of antigen-specific CD8^+^ T cells, which were evaluated by measuring the production of cytokines following peptide stimulation. In response to peptide challenge, most antigen-specific CD8^+^ T cells expressed granzyme B and CD107a with IFN-γ, establishing the cytotoxic capability of cyclin D1-specific CD8^+^ T cells. Antigen-specific CD4^+^ T cells also could expand via this prototype vaccine. Thus, our results demonstrated that targeting cyclin D1 to DCs could efficiently induce and activate cyclin D1-specific T cells.

Taken together, these approaches will facilitate the development of a novel DC vaccine for MCL. Mounting a potent cellular immune response in MCL patients is expected to bring better clinical benefits to patients.

## Materials and methods

### Study subjects

Five healthy donors and five MCL patients were studied. Their demographics and HLA types are listed in Table 1. All MCL patients are cyclin D1 positive. Apheresis and blood draws were obtained according to IRB-approved protocol (002–108) at Baylor Research Institute (Dallas, TX). All donors signed informed consent forms. Peripheral blood mononuclear cells (PBMCs) were purified by Ficoll (Amersham Biosciences, Pittsburgh, PA) density gradient centrifugation and cryopreserved until use. Total T cells or CD8^+^ T cells were enriched by negative selection following manufacture protocols with an EasySep Human T cell Enrichment kit or EasySep Human CD8^+^ T cell Enrichment kit (Stem Cell Technologies Inc.) to purity ≥98%.

### Peptide synthesis

The overlapping 15-mer cyclin D1 peptide library (Table 2) was staggered every four amino acids along the entire cyclin D1 sequence and generated at Mimotopes (Clayton, Australia). Peptides were dissolved in 5% acetonitrile (Sigma) at 10 mM and stored at −80°C.

### Media and reagents

Complete culture medium (CM) consisted of RPMI 1640 medium (Invitrogen, Carlsbad, CA), 1% L-glutamine (Sigma), 1% penicillin/streptomycin (Sigma), 50 mM 2-mercaptoethanol (Sigma), 1% sodium pyruvate (Sigma), 1% nonessential amino acids (Sigma), and 10% heat-inactivated FBS (fetal bovine serum, GIBCO). For T cell cultures, FBS was replaced by 10% heat-inactivated human serum AB (Gemcell). IL-2 (Genzyme) was used at 100 IU/ml. FITC mouse anti-human cyclin D1 antibody (G124-326) was purchased from BD Pharmingen.

### Cell lines

Granta 519 (mantle cell lymphoma cell line), K562, and T2 (HLA-A2-positive cell line) cells were purchased from the American Type Culture Collection (Manassas, VA). Cell lines were cultured in CM.

### Intracellular cytokine assay

Cultured PBMCs were restimulated with individual cyclin D1 15-mer peptides for 2 h. Then, Golgi-plug (BD Pharmingen) was added to the cultures and followed by another 4-h culture. After a total 6 h of stimulation, cells were harvested, surface stained with CD4 and CD8 mAbs, then fixed and permeabilized with Cytofix/Cytoperm solution (BD). Finally, the cells were stained intracellularly with anti-IFN-γ mAb (BD Pharmingen). The cells were acquired on Canton II or LSRII flow cytometer (BD Bioscience, San Jose, CA) and analyzed using FlowJo software (Treestar, Ashland, OR). When cultured T cells were analyzed, IFN-DCs were first loaded with cyclin D1 15-mer peptides for 1 h and then used to stimulated T cells.

### Peptide binding assay

The human TAP-deficient HLA-A*0201^+^ T2 cell line was used to measure the binding ability of cyclin D1 peptides to HLA-A*0201 molecules as described previously [54]. Briefly, 1 × 10^5^ T2 cells per well were incubated in a 96-well plate with or without individual peptides at a concentration of 25 μg/ml overnight. Then the cells were harvested, washed twice with FACS buffer, and stained with a PE-conjugated anti-HLA-A2 antibody (BB7.2; BD Pharmingen, San Diego, CA). The mean fluorescence intensity of HLA-A2 staining was analyzed by LSRII.

### Analysis of T cell responses by analysis of cytokine release

T2 cells were pre-loaded with 10-μM peptides for 2 h, washed with PBS twice, then cultured with effector cells at 1:1 ratio in a total volume of 200 μl medium with PMA (phorbolyristate acetate, 100 ng/ml). Culture supernatants were harvested 36 h later and tested for IL-2, IFN-γ, and IP-10 production via cytokine multiplex analysis.

### Preparation of killed MCL lymphoma cells

A 2 × 10^5^ cells/ml of the MCL cell line Granta 519 was treated with Velcade (Bortezomib, LC Laboratories) at 0.2 μg/ml for 17 h at 37°C. The obtained killed Granta 519 cells, a mixture of apoptotic and necrotic cells, were prepared in batches and frozen and stored in liquid nitrogen. Annexin V and propidium iodide (PI) staining was used to measure death of the lymphoma cells.

### Generation of CTLs and cytotoxicity assay

IFN-DCs were generated from elutriated monocytes by culturing in CellGenix medium (CellGenix) supplemented with 100 ng/ml human granulocyte-monocyte colony-stimulating factor (GM-CSF, Berlex Laboratories Inc.) and 500 U/ml IFN-α (INTRONA, Schering Corp) for 3 days. IFN-DCs were loaded with killed Granta 519 cells in a 2:1 ratio for 6 h, then cultured with autologous enriched CD8^+^ T cells at a 1:25 ratio, and supplemented with IL-7 (10 IU/ml) and IL-2 (10 IU/ml) at day 3 and IL-2 only at the second week. T cells were restimulated on day 7. The CTL activity was measured in a standard 4-h ^51^C-release assay at day 14. Briefly, T2 cells were loaded with or without 10-μM peptide for 2 h. Target cells were labeled with ^51^Cr (NEN Life Science Products, Boston, MA) for 1 h, washed then co-cultured with CTLs for 4 h. Specific lysis was calculated using the following formula: (where cpm is counts per minute): % release = 100 × (cpm experiment–cpm spontaneous release)/(cpm maximum release–cpm spontaneous release).

### Generation of recombinant fusion proteins

Antigen coding regions were transferred to vectors for stable transfection of CHO-S cell lines for expression and subsequent purification of anti-CD40-cyclin D1-pep and control hIgG4-cyclin D1-pep as described previously [55]. The control hIgG4 H chain variable and constant region was gb|BC025985.1| residues 19–1437 with T778C, A780C, and CTG at 779–801 to GAA changes. The control hIgG4 L chain variable and constant region was derived from clone CS0DI041YP06 (Invitrogen). Two sets of antibody-antigen fusion proteins were produced, one with cyclin D1 (NP_444284.1) residues 1–48 appended to the H chain C-terminus (pepA) and the other with residues 49–295 appended to the H-chain C-terminus (pepB). Efficient expression of the prototype vaccines was only obtained when the cyclin D1 peptide regions were flanked by the glycosylated flexible linker sequences ASQTPTNTISVTPTNNSTPTNNSNPKPNPAS and ASTNGSITVAATAPTVTPTVNATPSAAAS [44].

### Accession codes of CD40-targeting antibody

The 12E12 hybridoma is ATCC PTA 9854. The chimeric CD4012E12 L and CD4012E12 H chain sequences are GenBank HQ738667 and HQ738666, respectively.

### Statistical analysis

The local median regression method was used to set up a positive cutting line for cytokine production. Median plus 5 multiplied median absolute deviation (MAD) was considered statistically significant [56]. Unless otherwise indicated, the value of median plus 5 MAD was shown.

## Additional files

Additional file 1: Table S1. HLA types of healthy donors. Additional file 2: Figure S1. Cyclin D1-induced T cell responses in a healthy donor. PBMCs isolated from a healthy donor (ND239, HLA-A*0201*01 B*08*51/78 C*07 DRB1*0301*11 DQB1*02*03) were stimulated with the overlapping 15-mer cyclin D1 peptide library. Median plus 5 multiplied MAD is considered as a positive cutting line. (A) Supernatants were harvested to test cytokine secretion after 48-h co-culture. NoP is a no peptide negative control. (B) PBMCs were stimulated with cyclin D1 for 8 days and rested in serum-free medium for 3 days before boosting by the same peptide. Intracellular staining of IFN-γ was performed 6 h later. The cells without peptide were used as a negative control. Percentage of CD4^+^IFN-γ^+^ and CD8^+^IFN-γ^+^ population from IFN-γ intracellular staining was shown in a two-line graph. Medium plus 5 multiplied MAD is considered as a positive cut-off line (shown as a red dash line for CD4^+^ T cells and a blue dash line for CD8^+^ T cells).
