# Supplementary material for: A novel vaccine for mantle cell lymphoma based on targeting cyclin D1 to dendritic cells via CD40
Source: J Hematol Oncol. 2015 Apr 14;8:35. doi: 10.1186/s13045-015-0131-7 (PMC4424584; doi:10.1186/s13045-015-0131-7)
Supplement: Additional file 1: Table S1. — HLA types of healthy donors. [file 13045_2015_131_MOESM1_ESM.docx]

**Supplement Table 1. HLA types of healthy donors**

| Healthy Donor ID | HLA type |
| --- | --- |
| ND216  ND219  ND239  ND257  ND226 | A*0201*01 B*08*40 C*07*15 DRB1*01*14 DQB1*03*0501  A*0201*24 B*18*52 C*1202*1203 DRB1*11*15 DQB1*03*0602  A*0201*01 B*08*51/78 C*07 DRB1*0301*11 DQB1*02*03  A*0201*2902 B*18*40 C*03*07 DRB1*08*11 DQB1*0301*0402  A*0101*0301 B*0801*3501 C*0401*0701 DRB1*1302*1501 DQB1*0602*0604 |
