# Supplementary figures and images for: A novel vaccine for mantle cell lymphoma based on targeting cyclin D1 to dendritic cells via CD40
Source: J Hematol Oncol. 2015 Apr 14;8:35. doi: 10.1186/s13045-015-0131-7 (PMC4424584; doi:10.1186/s13045-015-0131-7)

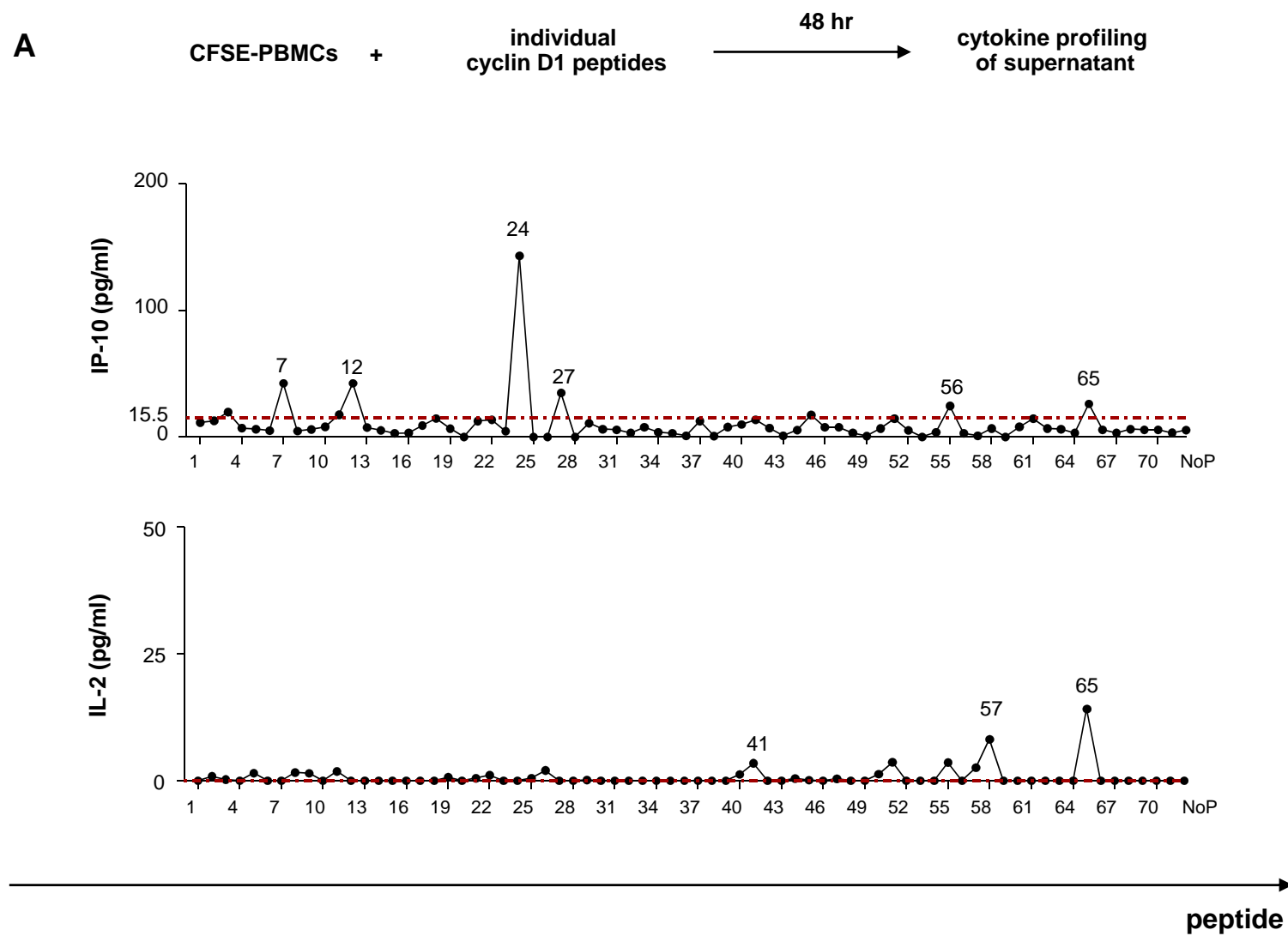

Supplement Figure S1

**B**

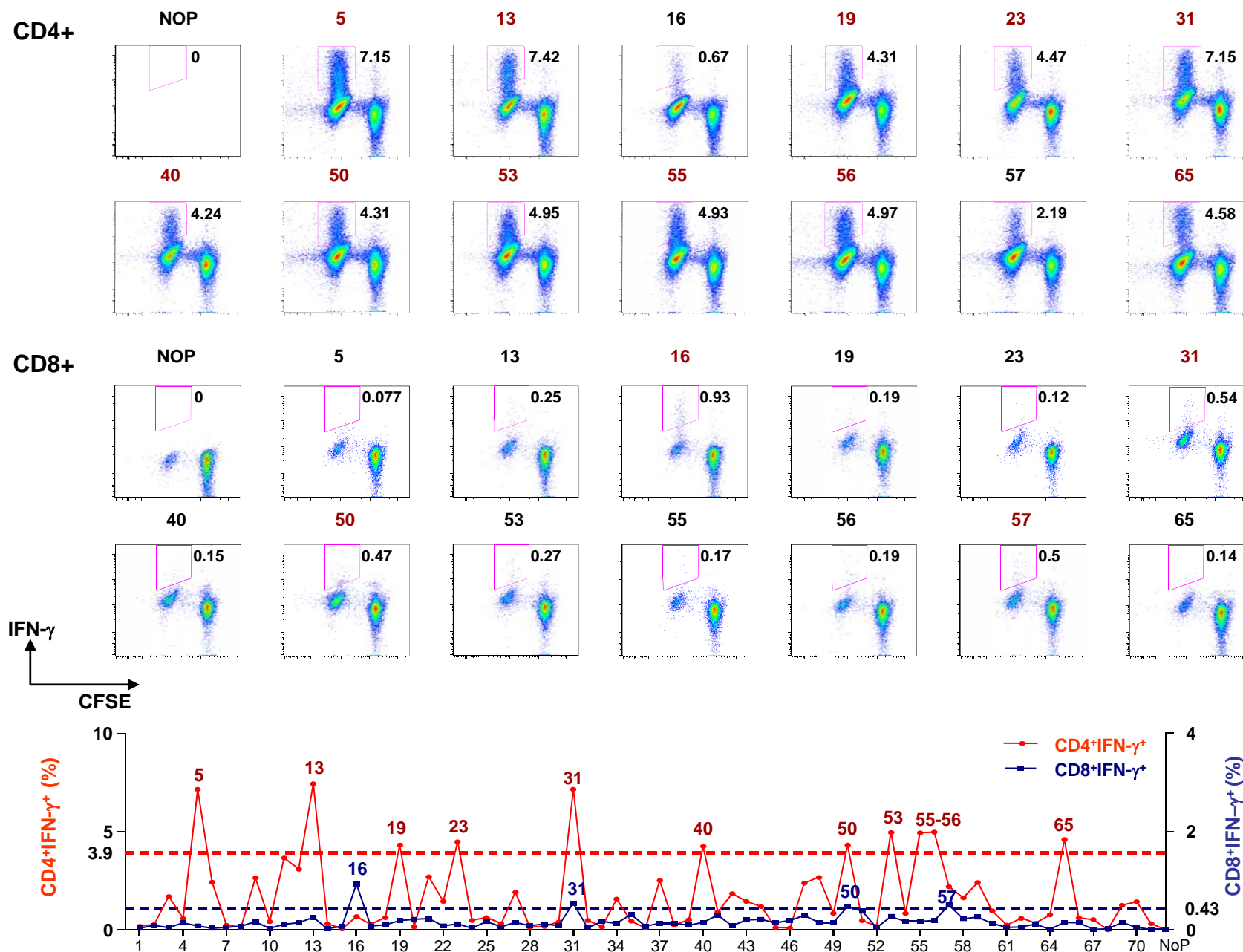

**Supplementary Figure S1 (B)**

Supplement: Additional file 2: Figure S1. — Cyclin D1-induced T cell responses in a healthy donor. PBMCs isolated from a healthy donor (ND239, HLA-A*0201*01 B*08*51/78 C*07 DRB1*0301*11 DQB1*02*03) were stimulated with the overlapping 15-mer cyclin D1 peptide library. Median plus 5 multiplied MAD is considered as a positive cutting line. (A) Supernatants were harvested to test cytokine secretion after 48-h co-culture. NoP is a no peptide negative control. (B) PBMCs were stimulated with cyclin D1 for 8 days and rested in serum-free medium for 3 days before boosting by the same peptide. Intracellular staining of IFN-γ was performed 6 h later. The cells without peptide were used as a negative control. Percentage of CD4+IFN-γ+ and CD8+IFN-γ+ population from IFN-γ intracellular staining was shown in a two-line graph. Medium plus 5 multiplied MAD is considered as a positive cut-off line (shown as a red dash line for CD4+ T cells and a blue dash line for CD8+ T cells). [file 13045_2015_131_MOESM2_ESM.pdf]
